# Association between Parkinson’s disease and the faecal eukaryotic microbiota

**DOI:** 10.1038/s41531-021-00244-0

**Published:** 2021-11-18

**Authors:** Severin Weis, Alexandra Meisner, Andreas Schwiertz, Marcus M. Unger, Anouck Becker, Klaus Faßbender, Sylvia Schnell, Karl-Herbert Schäfer, Markus Egert

**Affiliations:** 1 Faculty of Medical and Life Sciences, Institute of Precision Medicine, Microbiology and Hygiene Group, Furtwangen University, Villingen-Schwenningen, Germany; 2 MVZ Institute of Microecology, Herborn, Germany; 3 Department of Neurology, Saarland University, Homburg, Germany; 4 Institute of Applied Microbiology, Justus-Liebig-University, Giessen, Germany; 5 Working Group Enteric Nervous System (AGENS), University of Applied Sciences Kaiserslautern, Zweibrücken, Germany

**Keywords:** Next-generation sequencing, Systems biology, Parkinson's disease, Parkinson's disease, Gastrointestinal diseases

## Abstract

Parkinson’s disease (PD) is one of the most common neurodegenerative disease, and is so far not considered curable. PD patients suffer from several motor and non-motor symptoms, including gastrointestinal dysfunctions and alterations of the enteric nervous system. Constipation and additional intestinal affections can precede the classical motor symptoms by several years. Recently, we reported effects of PD and related medications on the faecal bacterial community of 34 German PD patients and 25 age-matched controls. Here, we used the same collective and analysed the V6 and V7 hypervariable region of PCR-amplified, eukaryotic 18S rRNA genes using an Illumina MiSeq platform. In all, 53% (18) of the PD samples and 72% (18) of the control samples yielded sufficient amplicons for downstream community analyses. The PD samples showed a significantly lower alpha and a different beta eukaryotic diversity than the controls. Most strikingly, we observed a significantly higher relative abundance of sequence affiliated with the *Geotrichum* genus in the PD samples (39.7%), when compared to the control samples (0.05%). In addition, we observed lower relative abundances of sequences affiliated with *Aspergillus/Penicillium*, *Charophyta/Linum*, unidentified Opisthokonta and three genera of minor abundant zooflagellates in the PD samples. Our data add knowledge to the small body of data about the eukaryotic microbiota of PD patients and suggest a potential association of certain gut eukaryotes and PD.

## Introduction

Parkinson’s disease (PD) represents the second most common human neurodegenerative disorder. So far, no treatment to stop the neurodegenerative process is available^1^. PD patients not only suffer from motor-associated and rare cognitive symptoms, but also from gastrointestinal (GI) symptoms^2^. GI-associated symptoms include constipation, prolonged intestinal transit time or defecation-associated dysfunctions and can precede the classical motor symptoms by several years^2–6^. Additionally, there is rising evidence that even the enteric nervous system (ENS) becomes compromised before the central nervous system^7–9^. These findings, alongside the growing numbers of published reports in this field, support the hypothesis that PD might begin, at least in a subgroup of patients, in the GI tract and propagate to the central nervous system^10,11^. The ENS is connected to the central nervous system via the sympathetic nervous system and the vagus nerve which form the so called gut–brain axis^12^. Experimental data suggest that this neuronal chain between ENS and the brain allows pathological peptides to propagate in a prion-like fashion between the gut and the brain, thereby modulating the course of this neurological disease^13–15^.

Gut-associated PD symptoms, such as constipation, are discussed to be dependent on changes of the intestinal microbiota composition and their metabolic activity^16^. Recent publications suggest that especially bacterial dysbiosis might play an important role in PD pathogenesis^17–20^. Other studies even indicate that the pathological process of PD alongside the gut–brain axis might be modulated or initiated by the gut microbiota^21,22^. Increased inflammation in PD, which is indicated by increased levels of faecal markers for inflammation and gut permeability, is suggested to be linked to dysbiosis in the gut environment^23,24^. Additionally, elevated levels of pathogenic or opportunistic pathogenic bacteria were found to be independent of medication^25^. The changes in relative abundance of some bacterial genera regarded as health promoting was found to be at least partially due to the medication^25^. To better understand the influence of the gut microbiota on the pathobiology of PD, and the influence of the disease on the microbiota itself, a deeper understanding of compositional changes in the gut microbiota is needed. So far, most PD microbiota studies focused on intestinal bacteria, and there is indeed growing evidence that bacterial dysbiosis may play an important role in PD pathogenesis^17–19^. However, the cause–effect relationship between PD pathogenesis and the structure and function of the gut microbiota is still obscure.

Besides bacteria, the gut microbiota also comprises archaea, viruses and various eukaryotic taxa including fungi. For bacteriophages, associations with shifts in the phagobiota in PD patients were reported, suggesting a closer look at the non-bacterial microbiota associated with this disease^26^. With estimated 0.01–0.1% of genes in stool samples, the gut mycobiome represents only a small fraction when compared to the faecal bacterial community^27^. Nevertheless, *Candida* species were reported to be present in about 70% of healthy adults^28^. The majority of intestinal eukaryotes still await cultivation and are difficult to identify^29^. However, the gut mycobiome was also reported to influence the gut–brain axis by secretion of neurotransmitters, modulation of cytokine production and production of short-chain fatty acids^30^. Additionally, reports showed a connection between gut eukaryotes and anorexia nervosa, a central nervous disease^31^, as well as autism spectrum disorders^32^.

In this study, we used next-generation sequencing to study differences in the faecal eukaryotic microbiota composition between a previously well studied group of PD patients and matched controls. To create a starting point and as only approximately 0.01% of human gut metagenomic sequences generated can be mapped to fungal genomes, a targeted, amplicon-based approach was chosen^27^. We analysed the same set of samples previously used by Unger et al. (2016) and Weis et al. (2019). Here, we used Illumina MiSeq-based next-generation sequencing to sequence the V6 and V7 region of the eukaryotic 18S rRNA gene^20,33^. Monitoring differences in the composition of faecal eukaryotes between PD patients and suitable healthy controls is a first step to elucidate whether gut eukaryotes mighty play any role in PD. Our study might help identifying organisms showing an association with PD and thereby increase our knowledge on the etiopathogenesis of PD.

## Results

### Sequencing and bioinformatics

**Fig. 1:**
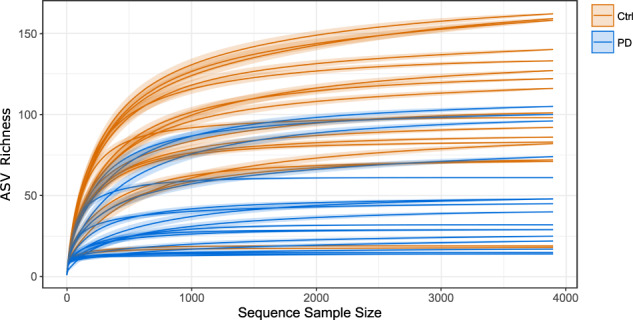
Alpha rarefaction curves to visualize the dependence of eukaryotic richness and sequencing depth. Shown are amplicon sequence variation (ASV) richness rarefaction curves for the sequencing sample depth for all samples. Blue: PD samples, orange: controls. Lines represent the mean of 50 sampling steps, each, with the opaque outlines as standard errors.

In all, 18 (53%) of the PD patient samples (6 females and 12 males) and 18 (72%) of the control group samples (10 females and 8 males) yielded sufficient 18S rRNA gene amplicons for downstream applications. Additional information about those samples is provided in Supplementary Table 1. The sequencing dataset comprised 493,979 partial 18S rRNA gene sequences after quality control, denoising, paired-end merging and chimera removal. The mean number of sequences per sample was 13,350 (min: 3895; max: 23,080). Assignment of taxonomy was done with the seven taxonomic ranks provided by SILVA, where the sixth rank equals genera for most listed taxa, the fifth lists mainly families, the fourth mainly orders, and so forth. Following rarefication to even depth of sequences per sample, 1612 Amplicon Sequence Variants (ASV) affiliated with 122 genera-equivalent ranks in the SILVA database, 95 family-equivalent ranks and 24 order equivalent ranks were assigned. ASV rarefaction curves (Fig. 1) showed that the sequencing depth was sufficient to detect the vast majority of taxa in all samples.

### Structural diversity measures

**Fig. 2:**
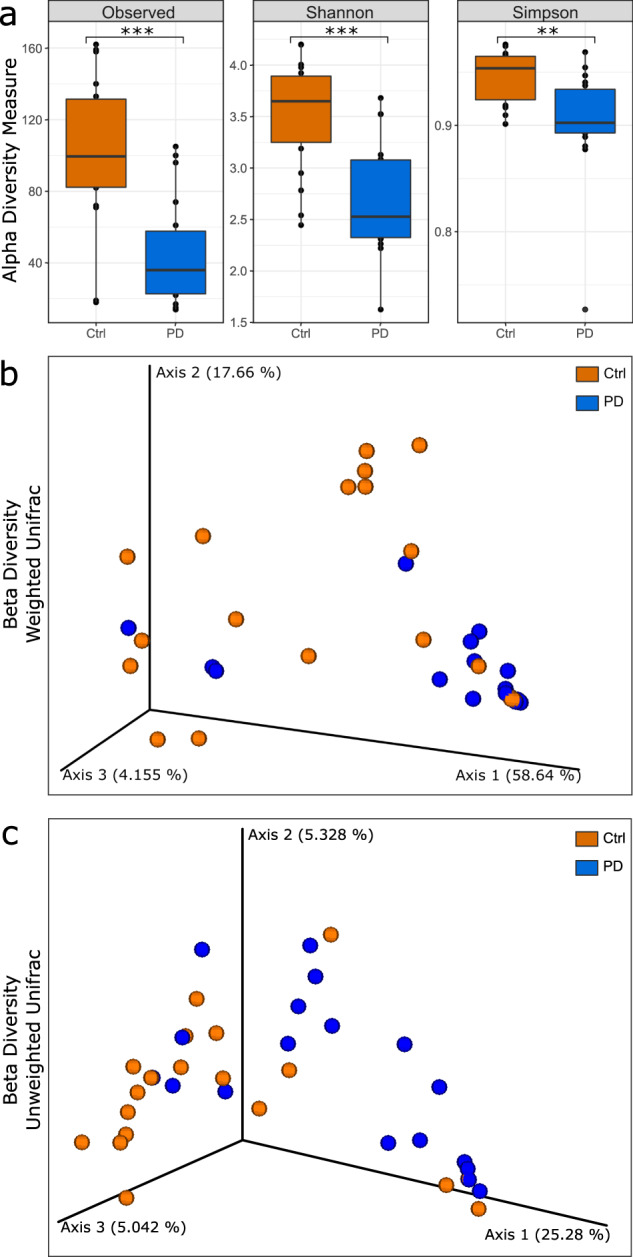
Alpha and beta-diversity plots for visualization of the eukaryotic community structure and according to differences between the control group and PD patients. PD samples are displayed in blue and control samples in orange. With Observed, Shannon and Simpson, the three most common used indices for alpha diversity are shown in (**a**). Alpha-diversity boxplots consist of the median and lower and upper quartiles while the whiskers represent the mininmal and maximal spread of individual samples. Significant differences between the groups are indicated with asterisks (***p < 0.001, **p < 0.01). For beta-diversity, PCoA plots of the weighted (**b**) and unweighted (**c**) UniFrac measures are shown. Both beta-diversity measures show significant differences between controls and PD.

The three applied indices for alpha diversity (Fig. 2a), Observed, Shannon and Simpson, revealed a significantly lower eukaryotic diversity in PD patients compared to the controls (Ctrl) (*p*_Observed_ < 0.002, *p*_Shannon_ < 0.001 *p*_Simpson_ < 0.002).

For beta-diversity, non-parametric multivariate analysis of variance (ADONIS) was calculated for the different groups (Fig. 2b, c). Between the PD and the control group, unity of fraction (UniFrac) measures revealed significant differences regarding the weighted (*p*_wUniFrac_ = 0.0073) and the unweighted (*p*_UniFrac_ < 0.0128) UniFrac measure. For the unweighted UniFrac measure, a significant difference was found between controls and PD patients treated with entacapone (*p*_UniFrac_ < 0.0483) or l-dopa (*p*_UniFrac_ < 0.0051) as well as between controls and PD patients without entacapone (*p*_UniFrac_ < 0.0012) or l-dopa (*p*_UniFrac_ < 0.0068) treatment. For the weighted UniFrac measure, a significant difference was found between PD patients without entacapone treatment and the controls (*p*_wUniFrac_ < 0.0003) as well as PD patients with entacapone treatment (*p*_wUniFrac_ < 0.0129). For the medication with l-Dopa, significant differences in the beta diversity were found between controls and both PD patients without treatment (*p*_wUniFrac_ < 0.0006) and those with treatment (*p*_wUniFrac_ < 0.0353).

### Compositional differences

Hypothesis testing revealed seven taxa on the genus-equivalent rank, assigned using the SILVA database, to be significantly different in relative abundance between PD and controls. The genera *Aspergillus* (*p*_FDR_ < 0.031)*, Cercomonas* (*p*_FDR_ < 0.037) and *Heteromita* (*p*_FDR_ < 0.022), as well as three unknown genus-equivalent features of the phylum division Charophyta (*p*_FDR_ < 0.019), the order Chromulinales (*p*_FDR_ < 0.013) and the clade Opisthokonta (*p*_FDR_ < 0.013), were found to be significantly lower in relative abundance in the group of PD patients when compared to the controls. In contrast, ASVs affiliated with the genus *Geotrichum* (*p*_FDR_ < 0.017) were significantly higher in relative abundance in the group of PD patients. As displayed in Table 1, the taxonomy assignment of the representative sequence with nucleotide-based basic local alignment search tool (BLASTN) for the nucleotide database provided by the National Center for Biotechnology Information (NCBI) revealed different species for those features. The genera *Geotrichum*, *Aspergillus, Cercomonas* and *Heteromita* were identified as being closely related (sequence identity >99%) to *Geotrichum candidum, Penicillium roqueforti, Paracercomonas* sp. and *Cercozoa* sp. B134, respectively. The highest degree of sequence identity (97–99%) for the unknown Charophyta taxon was found with *Linum usitatissimum* and with *Poterioochromonas malhamensis* in case of the unknown Chromulinales taxon. For Opisthokonta no sequences with identity >90% were found.

**Table 1:** Taxa differing significantly between PD samples and controls.

SILVA 132	“Phylum”	Opisthokonta	Archaeplastida	Opisthokonta	Opisthokonta	SAR	SAR	SAR
Assignment	“Class”	Nucletmycea	Chloroplastida	Nucletmycea	NA	Rhizaria	Stramenopiles	Rhizaria
	“Order”	Fungi	Charophyta	Fungi	NA	Cercozoa	Ochrophyta	Cercozoa
	“Family”	Dipodascaceae	NA	Aspergillaceae	NA	Cercomonadidae	Chromulinales	Glissomonadida
	“Genus”	*Geotrichum*	NA	*Aspergillus*	NA	*Cercomonas*	NA	*Heteromita*
NCBI nucleotide	Scientific name	*Geotrichum candidum*	*Linum usitatissimum*	*Penicillium roqueforti*		*Paracercomonas* sp.	*Poterioochromonas* *malhamensis*	*Cercozoa* sp. B134
MegaBLAST	Max score	573	555	564	No sequences	569	560	566
	Total score	573	1108	564	With identity	569	560	566
	Query cover	100%	100%	100%	>90%	99%	100%	100%
	*E* value	3.00E−159	1.00E−153	2.00E−156		3.00E−158	2.00E−155	4.00E−157
	% Identity	99.37%	98.72%	99.36%		99.68%	98.73%	99.05%
	Acc. length	1556	18299719	1775		1803	2517	1511
	Accession #	KU899094.1	CP027631.1	MT544459.1	FP929054.1	MG775632.1	MH536660.1	HQ918175.1
Statistical	Mean (Ctrl)	0.05%	12.24%	1.83%	2.97%	0.32%	0.81%	0.31%
Values	SE (Ctrl)	0.03%	2.36%	0.32%	0.40%	0.05%	0.10%	0.05%
	Mean (PD)	39.65%	3.07%	1.62%	0.61%	0.09%	0.08%	0.07%
	SE (PD)	5.57%	0.99%	0.50%	0.12%	0.02%	0.02%	0.02%
	*P* value	0.0004	0.0006	0.0015	0.0002	0.0021	0.0001	0.0009
	*P* (FDR)	0.0161	0.0182	0.0303	0.0121	0.0363	0.0121	0.0218
	ANCOM-II	TRUE	FALSE	FALSE	FALSE	FALSE	FALSE	FALSE

Shown are taxa that differed significantly between control (Ctrl) and Parkinson´s disease (PD) samples together with the annotation of the representative sequences based on the SILVA database entry in QIIME2 (SILVA) and the NCBI database using megablast. For each taxon, mean relative abundance (abun.), standard deviation (SD) and prevalence per subgroup are displayed, too. *P* values for hypothesis testing between PD and control samples with the two-sided Wilcoxon–Mann–Whitney test for unpaired and non-normally distributed samples (*p* value Ctrl vs. PD) were subjected to false discovery correction for multiple testing (FDR). NA not assigned.

Using analysis of composition of microbiomes (ANCOM-II^34^) with the non-condensed ASV table, all 12 ASVs assigned to *Geotrichum* were found to be significantly different between controls and PD patients (*W* > 0.8, *p*_FDR_ < 0.05). All of those ASV were then identified as *Geotrichum candidum* (sequence identity >98.7%) using BLASTN with the NCBI database. However, no other ASV was found to be significantly differing between PD patients and controls according to ANCOM-II, even with a *W*-cut-off at 0.6.

Samples from PD patients with elevated levels of faecal calprotectin (*n* = 6) showed no significant differences in community composition when compared to the controls or PD patients with normal calprotectin levels (*n* = 12). PD patients treated with l-dopa (*n* = 12) showed significantly higher relative abundances of *Geotrichum* (*p*_FDR_ = 0.0138). However, there were no significant differences between PD patients who were not treated with l-dopa (*n* = 6) and the control group or the PD patients treated with l-dopa. PD patients treated with Entacapone in addition to l-dopa (*n* = 7) also showed significant higher shares of *Geotrichum* when compared to the controls (*p*_FDR_ = 0.0069). Finally, no significant differences were found between PD patients without Entacapone treatment (*n* = 11) and the control group, or between PD patients without Entacapone treatment and PD patients treated with Entacapone.

Correlation analysis using Spearman rho and Pearson rho did not reveal any significant correlation between the count data of the *Geotrichum*-affiliated sequences and the count data of any bacterial genera from the same samples, investigated in our previous study^20^.

## Discussion

Several publications addressed the composition of the intestinal bacterial microbiota in PD patients by means of qPCR^33^ or sequencing of different regions of 16S rRNA gene amplicons, using pyrosequencing^21,35^, Ion Torrent^20^ and Illumina MiSeq technology^20,36,37^. However, only little is known about associations and potential functional implications of the eukaryotic gut microbiota with PD. Recently, sequencing of fungal-specific internal transcribed spacer (ITS)-2 amplicons, derived from faecal samples of a cohort of PD patients, did not reveal any genetic differences in relative abundance or diversity between PD and controls^38^. The authors also reported a very low fungal load for their samples and significantly lower amounts of fungal DNA in PD patients^38^. This finding might partly explain why 28% (controls) and 47% (PD) of our samples did not yield sufficient amplicons for downstream analyses. However, we found significant differences in relative abundance for several eukaryotic taxa, including fungi, between the remaining PD and control samples.

Our data indicate a significantly decreased richness of eukaryotes according to the observed richness, Simpson metric and Shannon metric (Fig. 2a). This is in contrast to Cirstea et al.^38^ who did not report changes in richness, which might be due to fundamental differences in the sample groups, such as dietary habits, geographic origin or medical history and status. However, our finding matches previously published decreases in bacterial richness^20,36,39^. The beta diversity measures also showed significant differences between PD and control samples for the unweighted and weighted UniFrac metric (Fig. 2b, c), suggesting substantial differences in community composition between the eukaryotic microbiota of PD patients and the healthy control group. Therefore, our expectation was to find significant differences in the relative abundance of several taxa.

For the bacterial community of this study cohort, we previously reported a significantly lower observed alpha diversity under PD, too^20^. However, in contrast to the eukaryotic data presented here, indicators assuming uniformity of the distribution (Shannon and Simpson) and all beta-diversity parameters did not show significant differences between controls and PD patients^20^, suggesting that eukaryotic and prokaryotic diversity might behave differently under PD. However, these differences might also be due to the smaller sample size analysed here or the use of different sequencing methods.

Even though we found seven genus-equivalent taxa being significantly different in their relative abundance between control and PD samples, only *Geotrichum* was found to be significantly different when using ANCOM-II, i.e., when using a compositional approach^34^. It might be speculated that the observed depletion of the other, minor abundant groups is just due to the overgrowth of *Geotrichum* in the PD samples rather than actual depletion. Further studies might reveal whether there are significant differences in other groups, such as protozoa too. Our data at least suggest that besides moulds like *Geotrichum* also other eukaryotic genera might differ between PD patients and controls. Although bacterivorous gliding zooflaellates such as *Cercomonas (Paracercomonas), Heteromita (Cercozoa* sp. B134) and *Poterioochromonas* are not regarded as typical representatives of the human gut microbiota, grazing of protists is often selective and migth influence the virulence, metabolism and morphology of intestinal bacteria^40–43^. *Penicillium* and *Aspergillus* are commonly found in the healthy human gut^44^. Moulds are known to produce various mycotoxins and to influence the mucosal cytokine response, which affect GI homoeostasis and the composition of the bacterial gut microbiota^45,46^. Finally, some differences in relative abundance of eukaryotic taxa such as *Linum usitatissimum* or *Penicillium roqueforti* might just result from differential nutrition.

In contrast to the other genera, the family Dipodascaceae and its affiliated genus *Geotrichum* showed a markedly higher relative abundance in the PD samples compared to the controls. Dipodascaceae are commonly found in human stool samples and are potentially able to colonize the gut^44^. Few species are regarded as a health issue and capable of causing geotrichosis, including *Geotrichum candidum*, which produces several toxins^47^. However, this species is also used in dairy production^48^, where it is also regarded as spoilage organism^48^ causing opportunistic infections in immunocompromised patients^49^. An increased abundance of lactococcal phages, which are also present in dairy products, was suggested to cause depletion of *Lactococcus* in PD patients^26^. Together with our data, such observations suggest a special influence of dietary habits, in particular dairy products, on the intestinal microbiota of PD patients. It might be also be speculated that PD patients offer more suitable intestinal conditions for this species than healthy patients do. Due to its marked and significant differences in relative abundance between controls and PD patients, *Geotrichum* was used for a correlation analysis with our previously published bacterial 16S rRNA gene data^20^. However, after false discovery rate correction no significant correlation with any bacterial genus was found, suggesting no direct fungus–bacterium associations here. However, also technological reasons (e.g. the differing sequencing technologies) or the time gap between the studies might at least partially explain these missing correlations.

Clearly, the respective subsample sizes in our study were small, also due to the discarded samples, so that all hypotheses need to be verified. Additionally, some findings might also be caused by different food preferences of the investigated patients, although all of them reported an omnivorous European diet. Clearly, further analyses are needed to verify whether the observed differences in community composition might be of biological/medical relevance in the case of PD. To do so, more complex analyses using multiple variable regions of the 18S rRNA gene sequence and ITS region sequences or even a metagenomics approach will be needed^50^. Since only 0.01% of metagenomics sequences generated from human gut samples can be aligned to fungal genomes, such metagenomics approaches require sufficient sequencing depth^27^.

We were able to show that PD faecal samples contained a eukaryotic microbial community of lower diversity and that several eukaryotic taxa differed in relative abundance between PD and control faecal samples, suggesting an association of PD and the eukaryotic microbiota. Within the PD group, we particularly observed a very high relative abundance of *Geotrichum*, a fungal genus commonly found in the human gut and usually not regarded as an intestinal pathogen. In addition, difference in a few minor abundant groups were detected. Future studies will have to show, whether these findings are reproducible, of functional relevance or diagnostic value. Despite sampling issues (sample age, small sample size), environmental factors, such as geographic and cultural background of the studied cohort and the used sequencing techniques might explain differences to previous studies. Clearly, studies with larger patient and control cohorts are needed for deeper insights into the potential links between gut eukaryotes and PD, ideally combing metagenomic, metatranscriptomic and/or metabolomic approaches.

## Methods

### Cohort, sample collection and DNA isolation

All 34 PD patients (10 females, 24 males), whose faecal samples were used in this work, were diagnosed according to the UK PD Society Brain Bank Clinical Diagnostic Criteria^51^. The 25 control persons (14 females, 11 males) were age matched and did not report any pre-existing medical conditions or medication. All subjects followed an omnivorous diet without special dietary habits, and did not report on acute or chronic gastrointestinal disorders or intake of antibiotics, pro- or prebiotics up to 3 months prior to sampling. For more sample details, sample collection and DNA isolation, the reader is referred to our previous publication on the bacterial community composition of the same study cohort^20^. In short, subjects were provided with sterile faecal collector sets (MED AUXIL, Süsse Labortechnik, Gudensberg, Germany) for home sampling. Samples were stored and transported at least at −20 °C and DNA was isolated using the FastDNA SPIN kit for faeces (MP Biomedicals, Heidelberg, Germany). The study was approved by the ethics committee of the medical association of the German federal state of Saarland and is recorded therein with the identification number 111/12. All enrolled subjects provided written informed consent for their participation.

### Library preparation and sequencing

Sequencing library preparation of the V6 and V7 region of eukaryotic 18S rRNA genes were performed using the 18S-specific primers 1152F (5′- TGAAACTTRAAGRAATTGACGGA-3′) and 1428R (5′-GGRCATMACDGACCTGYTAT-3′)^27^ with additional adapter sequences for the Illumina Nextera indexing to produce amplicons of approximately 250–380 bp length. Replicate PCR amplification per sample, amplicon verification and purification were performed twice using the KAPA HiFi Polymerase-Kit (Roche, Mannheim, Germany) as described elsewhere^52^. Fifty microliters PCRs were set up with a 5 µl DNA template, 30 amplification cycles (initial denaturation: 3 min, 95 °C; 30 cycles: 30 s, 95 °C, 30 s, 55 °C, 45 s, 72 °C; final elongation: 5 min, 72 °C), *Saccharomyces cerevisiae* DNA as positive controls, and water-template controls. Verification of amplification by agarose gel electrophoresis using Midori Green as DNA-dye (Biozym, Olderndorf, Germany) showed that 18 control samples and 18 PD samples produced sufficient amplicons for downstream analyses. Metadata for these remaining samples is provided in Supplementary Table 1. Successful replicate PCR reactions of the same samples were pooled and purified using Agencourt AMPure beads (Beckman Coulter, Krefeld, Germany).

Unique index barcodes with sequencing adaptors were added to the amplicon targets as described previously^52^ using the unique indexing primer combinations of the Nextera XT index kit v2 set D (Illumina), 5 µl of purified template, and the PCR profile detailed above, albeit with 8 cycles and a total volume of 25 µl. After another purification step, quality checks, checks for library size and DNA concentration using Agilent DNA 1000 chips in the Agilent bionalyzer (Agilent Technologies, Waldbronn, Germany) and the Qubit dsDNA HS Assay Kit (Thermo Fisher Scientific, Schwerte, Germany) were carried out as described before^52^; libraries were normalized to 4 nM and pooled for sequencing. By following the manufacturer’s instructions, the pooled libraries were sequenced in a final concentration of 6 pM on an Illumina MiSeq platform (Illumina). For the targeted amplicon sequencing procedure, a 600 cycle paired-end format (2 × 300 bp + 2 × 8 bp index cycles) was chosen using the MiSeq Reagent Kit v3 with 20% phiX spike in in the pooled library.

### Bioinformatics and statistics

Sequence data were processed using QIIME 2 version 2019.7 (ref. ^53^). Quality cut-offs were performed with the median at *Q* ≥ 25. Minimum and maximum sequence lengths of 200 and 1000 bp were used (QIIME2 default). Denoising, merging paired-ends and removal of chimeras were performed using the DADA2 pipeline of QIIME2. Amplicon sequence variations (ASV) were chosen within 99% sequence identity. A primer-fitted taxonomy classifier, trained with the SILVA database release 132 using the classify-sklearn algorithm in QIIME2, was used to assign taxonomy and align sequences^54^.

Using a QIIME2-generated ASV table, normalized by rarefaction to even depth, alpha-diversity indices for Observed, Shannon and Simpson metrics, as well as beta-diversity indices for weighted and unweighted UniFrac were calculated with the according QIIME2 plugins. PCoA plots were also produced with QIIME2 using the previously calculated distance matrices. Hypothesis testing of the alpha- and beta-diversity measures were also performed in QIIME2 using Kruskal–Wallis statistics for alpha diversity and PERMANOVA with 10,000-fold permutations for beta-diversity measures.

Further statistical analyses were calculated within R (version 4.0.2). The phyloseq package (version 1.32.0) was used as interface between QIIME2 and R to prepare the previously rarefied table provided by QIIME2 by condensing ASVs onto the genus-equivalent rank and calculating alpha diversity boxplots with the QIIME2 provided rarefied table^55^. To find significant differences in feature counts between PD and controls, a two-sided Wilcoxon–Mann–Whitney test for unpaired and non-normally distributed samples in a 10,000-fold Monte-Carlo simulation within the R package coin (version 1.3.1) was used^56^. For compositional data analysis, the statistical framework “Analysis of Composition of Microbiomes” (ANCOM^57^) in the zero inflation avoiding version ANCOM-II^34^ was used in an ASV-wise manner with the non-condensed ASV table to compare compositional differences in the the Control and PD groups. Zero- and out- cut-off were kept at the default of 0.90 and 0.05, respectively. The FDR adjusted alpha-value was set as 0.05 and for the *W*-statistic cut-off the arbitrary numbers 0.7 and 0.8 were used. All *p* values from hypothesis testing were corrected for the false discovery rate (FDR) in their corresponding group^58^.

Correlation analysis for Spearman correlation index and Pearson correlation of the merged count data of the *Geotrichum* genus with all bacterial genera published before^20^ of the same samples successfully sequenced in this work. Correlations were calculated using the cor.test function of the stats package in R^59^. Additionally, correlations between *Geotrichum* and bacterial genera were calculated for the PD group only.

For all ASVs or ASV clusters differing significantly in relative abundance in the performed hypothesis testing, the representative sequence was used for further comparison with the National Center for Biotechnology Information (NCBI) nucleotide database. Here, the nucleotide-based basic local alignment search tool (BLAST) with the megablast option without further exclusions or limitations against the standard databases provided by NCBI was used.

### Reporting summary

Further information on research design is available in the Nature Research Reporting Summary linked to this article.

## Supplementary information

Supplementary Information

Reporting Summary

## Data Availability

Sequences generated and analysed here are accessible at the European Nucleotide Archive (ENA) under the accession number PRJEB45549. Subject metadata is included in the Supplementary Files. Complete, rarefied feature and taxonomy tables with provenance (qza-format), as well as other datasets are available from the corresponding author on reasonable request.

## Code availability

Only open source code from QIIME2 (2019.7) and the cited R (4.0.2) packages (phyloseq 1.32.0, coin 1.3.1) were used, using either the default settings or the settings stated in the “Methods” section. Further information for clarification is available from the corresponding author on reasonable request.
