# Supplementary material for: Association between Parkinson’s disease and the faecal eukaryotic microbiota
Source: NPJ Parkinsons Dis. 2021 Nov 18;7:101. doi: 10.1038/s41531-021-00244-0 (PMC8602383; doi:10.1038/s41531-021-00244-0)
Supplement: Supplementary file 1 — Supplementary Information [file 41531_2021_244_MOESM1_ESM.pdf]

Supplementary Table 1. Metadata for PD and controls samples that yielded sufficient 18S rRNA gene amplicons for downstream analyses.

SampleID	Group	Sex (biol.)	Age (month)	Disease duration (month)	Hoehn-Yahr stage	Phenotype	Calprotectin [µg/g]		Smoker	Appendektomy	PD in the family	Constipation	L-Dopa	Entacapone	*Geotrichum [rel. abundance]*
P001	PD	f	50	112	2.5	E	-	<20	no	yes	yes	no	+	-	0.0%
P002	PD	m	78	14	2	E	-	<20	no	yes	no	no	+	-	99.5%
P003	PD	m	65	139	3	HR	-	<20	no	no	no	no	+	+	24.4%
P009	PD	m	68	69	3	T	-	<20	no	yes	no	no	-	-	99.2%
P010	PD	f	74	108	2	HR	-	<20	no	no	yes	yes	+	+	98.4%
P011	PD	f	67	168	3	HR	+	65.63	no	no	yes	yes	+	+	0.2%
P012	PD	f	78	99	4	E	+	345.33	no	no	no	no	+	-	0.3%
P013	PD	m	66	159	4	E	+	568.18	no	no	no	no	+	+	0.0%
P014	PD	m	69	147	3	E	-	<20	no	no	yes	no	+	-	0.0%
P018	PD	m	53	38	2	HR	+	371.78	no	no	no	yes	-	-	98.8%
P019	PD	m	60	84	3	HR	-	41.616	no	no	yes	no	+	+	0.0%
P021	PD	m	78	84	1	HR	-	<20	no	yes	yes	no	-	-	8.3%
P025	PD	m	75	228	3	E	-	42.09	no	yes	no	no	+	+	0.0%
P026	PD	m	69	15	2	HR	-	<20	no	no	no	no	-	-	99.9%
P027	PD	m	53	14	2	E	-	<20	no	no	no	no	-	-	94.6%
P029	PD	f	62	17	2.5	T	+	107.46	yes	yes	no	no	-	-	0.0%
P033	PD	m	62	24	2	T	-	30.7	no	no	no	yes	+	-	90.2%
P036	PD	f	77	87	2	HR	+	96.5	no	no	no	yes	+	+	0.0%
PK043	Ctrl	f	63	-	-	-	-	<20	no	-	-	no	-	-	0.0%
PK044	Ctrl	m	66	-	-	-	-	<20	yes	-	-	no	-	-	0.0%
PK045	Ctrl	f	65	-	-	-	-	<20	no	-	-	no	-	-	0.0%
PK047	Ctrl	m	67	-	-	-	+	151	no	-	-	yes	-	-	0.0%
PK048	Ctrl	f	63	-	-	-	-	<20	yes	-	-	no	-	-	0.0%
PK049	Ctrl	m	72	-	-	-	-	<20	no	-	-	no	-	-	0.0%
PK050	Ctrl	f	70	-	-	-	-	<20	no	-	-	no	-	-	0.0%
PK051	Ctrl	f	63	-	-	-	-	<20	yes	-	-	no	-	-	0.0%
PK052	Ctrl	m	59	-	-	-	-	<20	yes	-	-	no	-	-	0.0%
PK053	Ctrl	f	57	-	-	-	-	<20	no	-	-	yes	-	-	0.7%
PK054	Ctrl	m	56	-	-	-	-	<20	no	-	-	no	-	-	0.0%
PK057	Ctrl	m	76	-	-	-	-	<20	no	-	-	no	-	-	0.0%
PK058	Ctrl	m	71	-	-	-	+	27.337	no	-	-	no	-	-	0.0%
PK059	Ctrl	f	74	-	-	-	-	<20	no	-	-	no	-	-	0.0%
PK060	Ctrl	f	66	-	-	-	-	<20	no	-	-	no	-	-	0.1%
PK061	Ctrl	m	70	-	-	-	-	<20	no	-	-	no	-	-	0.0%
PK062	Ctrl	f	64	-	-	-	-	<20	no	-	-	no	-	-	0.0%
PK063	Ctrl	f	64	-	-	-	-	<20	no	-	-	no	-	-	0.0%

PD	Patients with Parkinson's disease	Ctrl	healthy control persons
m	male	f	female
E	equivalent pänotype	+	positve / with named medication
HR	hypokinetic-rigid phenotype	-	negative / without named medication
T	tremor dominant phenotype		

The relative abundance of *Geotrichum* is displayed as percent of the rarefied sequences for each sample

Supplementary Table 2. Mean abundances with standard error (SE) and FDR corrected p-values for all genus-rank equivalent ASVs.

Phylum	Class	Order	Family	Genus	$Mean_{Ctrl}$	SE_{Ctrl}	$Mean_{PD}$	SE_{PD}	P	P_{FDR}	
Opisthokonta	*Nucletmycea*	*Fungi*	*Dipodascaceae*	*Geotrichum*	0.05%	0.03%	39.65%	5.57%	0.0004	0.0161	*
Archaeplastida	*Chloroplastida*	*Charophyta*	*NA*	*NA*	12.24%	2.36%	3.07%	0.99%	0.0006	0.0182	*
Opisthokonta	*Nucletmycea*	*Fungi*	*Aspergillaceae*	*Aspergillus*	1.83%	0.32%	1.62%	0.50%	0.0015	0.0303	*
Opisthokonta	*NA*	*NA*	*NA*	*NA*	2.97%	0.40%	0.61%	0.12%	0.0002	0.0121	*
SAR	*Rhizaria*	*Cercozoa*	*Cercomonadidae*	*Cercomonas*	0.32%	0.05%	0.09%	0.02%	0.0021	0.0363	*
SAR	*Stramenopiles*	*Ochrophyta*	*Chromulinales*	*NA*	0.81%	0.10%	0.08%	0.02%	0.0001	0.0121	*
SAR	*Rhizaria*	*Cercozoa*	*Glissomonadida*	*Heteromita*	0.31%	0.05%	0.07%	0.02%	0.0009	0.0218	*
Opisthokonta	*Nucletmycea*	*Fungi*	*Debaryomycetaceae*	*NA*	18.96%	5.53%	14.80%	4.02%	0.2348	0.8252	
NA	*NA*	*NA*	*NA*	*NA*	34.42%	4.61%	13.38%	2.65%	0.0042	0.0635	
Opisthokonta	*Nucletmycea*	*Fungi*	*Sporidiobolaceae*	*Rhodotorula*	0.04%	0.02%	9.65%	3.28%	0.7924	1	
Opisthokonta	*Nucletmycea*	*Fungi*	*Saccharomycetaceae*	*NA*	9.88%	2.40%	6.12%	2.25%	0.0379	0.3025	
Opisthokonta	*Nucletmycea*	*Fungi*	*NA*	*NA*	3.47%	1.05%	5.57%	2.46%	0.0078	0.0891	
Opisthokonta	*Nucletmycea*	*Fungi*	*Aspergillaceae*	*NA*	0.81%	0.22%	2.46%	0.82%	0.1637	0.8252	
Opisthokonta	*Holozoa*	*Metazoa (Animalia)*	*Tetrapoda*	*Mammalia*	4.95%	1.33%	1.41%	0.38%	0.0054	0.0726	
Archaeplastida	*Chloroplastida*	*Charophyta*	*Lamiales*	*NA*	0.41%	0.18%	0.23%	0.06%	0.9046	1	
Archaeplastida	*Chloroplastida*	*Charophyta*	*Poales*	*NA*	0.45%	0.15%	0.23%	0.06%	0.2387	0.8252	
Archaeplastida	*Chloroplastida*	*Charophyta*	*Brassicales*	*NA*	2.16%	1.50%	0.17%	0.07%	0.4531	1	
Opisthokonta	*Nucletmycea*	*Fungi*	*Malasseziaceae*	*Malassezia*	0.25%	0.05%	0.10%	0.02%	0.0438	0.3025	
Archaeplastida	*Chloroplastida*	*Charophyta*	*Asparagales*	*Allium*	0.04%	0.02%	0.10%	0.05%	1	1	

Phylum	Class	Order	Family	Genus	$Mean_{Ctrl}$	SE_{Ctrl}	$Mean_{PD}$	SE_{PD}	P	P_{FDR}	
Archaeplastida	*Chloroplastida*	*Charophyta*	*Fabales*	*NA*	0.13%	0.07%	0.07%	0.01%	0.6941	1	
SAR	*Rhizaria*	*Cercozoa*	*Spongomonadida*	*Spongomonas*	0.08%	0.02%	0.04%	0.01%	0.2646	0.8894	
Opisthokonta	*Holozoa*	*Metazoa (Animalia)*	*NA*	*NA*	0.31%	0.07%	0.04%	0.01%	0.0081	0.0891	
Archaeplastida	*Chloroplastida*	*Charophyta*	*Zingiberales*	*NA*	0.13%	0.05%	0.04%	0.02%	0.3547	1	
Opisthokonta	*Holozoa*	*Metazoa (Animalia)*	*Neoptera*	*Diptera*	0.04%	0.01%	0.04%	0.02%	0.1956	0.8252	
Excavata	*Discoba*	*Discicristata*	*Tetramitia*	*Naegleria*	0.10%	0.02%	0.03%	0.01%	0.0935	0.5608	
Archaeplastida	*Chloroplastida*	*Charophyta*	*Asparagales*	*NA*	0.00%	0.00%	0.03%	0.01%	1	1	
Excavata	*Discoba*	*Discicristata*	*Neobodonida*	*Rhynchomonas*	0.01%	0.01%	0.02%	0.01%	0.8663	1	
Opisthokonta	*Nucletmycea*	*Fungi*	*Pleosporaceae*	*Alternaria*	0.02%	0.01%	0.02%	0.01%	0.5928	1	
Opisthokonta	*Nucletmycea*	*Fungi*	*Mucoraceae*	*Mucor*	0.10%	0.03%	0.02%	0.01%	0.0393	0.3025	
Opisthokonta	*Nucletmycea*	*Fungi*	*Tilletiaceae*	*Tilletia*	0.10%	0.03%	0.02%	0.01%	0.1004	0.5608	
Opisthokonta	*Nucletmycea*	*Fungi*	*Lichtheimiaceae*	*Lichtheimia*	0.01%	0.00%	0.02%	0.01%	1	1	
Archaeplastida	*Chloroplastida*	*Charophyta*	*Poales*	*Oryza*	0.00%	0.00%	0.02%	0.01%	1	1	
Excavata	*Metamonada*	*Parabasalia*	*Tritrichomonadea*	*Dientamoeba*	0.00%	0.00%	0.02%	0.01%	1	1	
SAR	*Alveolata*	*Ciliophora*	*Colpodida*	*NA*	0.07%	0.03%	0.02%	0.01%	0.2260	0.8252	
SAR	*Stramenopiles*	*Incertae Sedis*	*Blastocystis*	*Blastocystis sp. CK92-4*	0.00%	0.00%	0.02%	0.01%	1	1	
Opisthokonta	*Nucletmycea*	*Fungi*	*Boletaceae*	*Boletus*	0.00%	0.00%	0.02%	0.01%	1	1	
SAR	*Stramenopiles*	*Peronosporomycetes*	*Phytophthora*	*NA*	0.06%	0.02%	0.01%	0.00%	0.2755	0.9010	
Opisthokonta	*Holozoa*	*Metazoa (Animalia)*	*Arachnida*	*Araneae*	0.03%	0.01%	0.01%	0.00%	0.4021	1	
Cryptophyceae	*Goniomonas*	*uncultured microeukaryote*	*uncultured microeukaryote*	*uncultured microeukaryote*	0.00%	0.00%	0.01%	0.00%	1	1	

Phylum	Class	Order	Family	Genus	$Mean_{Ctrl}$	SE_{Ctrl}	$Mean_{PD}$	SE_{PD}	P	P_{FDR}	
Archaeplastida	*Chloroplastida*	*Charophyta*	*Malvales*	*NA*	0.13%	0.05%	0.01%	0.00%	0.0716	0.4560	
Excavata	*Discoba*	*Discicristata*	*Metakinetoplastina*	*Neobodonida*	0.00%	0.00%	0.01%	0.00%	1	1	
SAR	*Rhizaria*	*Cercozoa*	*Cercomonadidae*	*Eocercomonas*	0.09%	0.03%	0.01%	0.00%	0.0323	0.3006	
SAR	*Alveolata*	*Apicomplexa*	*Cryptosporida*	*Cryptosporidium*	0.14%	0.07%	0.01%	0.00%	0.2223	0.8252	
Archaeplastida	*Chloroplastida*	*Chlorophyta*	*Microthamniales*	*Dictyochloropsis*	0.00%	0.00%	0.01%	0.00%	1	1	
Archaeplastida	*Chloroplastida*	*Charophyta*	*Magnoliales*	*NA*	0.07%	0.04%	0.00%	0.00%	0.4855	1	
Archaeplastida	*Chloroplastida*	*Chlorophyta*	*NA*	*NA*	0.01%	0.00%	0.00%	0.00%	0.6074	1	
Opisthokonta	*Holozoa*	*Metazoa (Animalia)*	*Bdelloidea*	*NA*	0.00%	0.00%	0.00%	0.00%	1	1	
Opisthokonta	*Nucletmycea*	*Fungi*	*Russulales*	*uncultured*	0.00%	0.00%	0.00%	0.00%	1	1	
Opisthokonta	*Holozoa*	*Choanoflagellida*	*Craspedida*	*Salpingoecidae*	0.01%	0.01%	0.00%	0.00%	0.3636	1	
SAR	*Stramenopiles*	*Ochrophyta*	*Chromulinales*	*Spumella*	0.05%	0.04%	0.00%	0.00%	1	1	
Opisthokonta	*Nucletmycea*	*Fungi*	*Lichtheimiaceae*	*Rhizomucor*	0.00%	0.00%	0.00%	0.00%	1	1	
Opisthokonta	*Nucletmycea*	*Fungi*	*Incertae Sedis*	*Acremonium*	0.05%	0.04%	0.00%	0.00%	1	1	
Archaeplastida	*Chloroplastida*	*Chlorophyta*	*Trebouxiophyceae*	*Apatococcus lobatus*	0.12%	0.04%	0.00%	0.00%	0.0186	0.1876	
Archaeplastida	*Chloroplastida*	*Chlorophyta*	*Trebouxiophyceae*	*Prototheca zopfii var. hydrocarbonea*	0.13%	0.06%	0.00%	0.00%	0.0412	0.3025	
Opisthokonta	*Nucletmycea*	*Fungi*	*Rhizopodaceae*	*Rhizopus*	0.13%	0.05%	0.00%	0.00%	0.0450	0.3025	
Opisthokonta	*Holozoa*	*Metazoa (Animalia)*	*Arachnida*	*Acari*	0.02%	0.01%	0.00%	0.00%	0.1045	0.5608	
SAR	*Alveolata*	*Apicomplexa*	*NA*	*NA*	0.16%	0.07%	0.00%	0.00%	0.1066	0.5608	
Opisthokonta	*Nucletmycea*	*Fungi*	*Moniliellaceae*	*Moniliella*	0.02%	0.01%	0.00%	0.00%	0.2249	0.8252	
Opisthokonta	*Holozoa*	*NA*	*NA*	*NA*	0.04%	0.02%	0.00%	0.00%	0.2267	0.8252	

Phylum	Class	Order	Family	Genus	Mean$_{Ctrl}$	SE$_{Ctrl}$	Mean$_{PD}$	SE$_{PD}$	P	P$_{FDR}$	
Opisthokonta	*Nucletmycea*	*Fungi*	*Filobasidiaceae*	*NA*	0.06%	0.03%	0.00%	0.00%	0.2303	0.8252	
SAR	*Stramenopiles*	*Ochrophyta*	*Ochromonadales*	*Ochromonas*	0.07%	0.03%	0.00%	0.00%	0.2304	0.8252	
Opisthokonta	*Nucletmycea*	*Fungi*	*Microascaceae*	*NA*	0.08%	0.04%	0.00%	0.00%	0.2328	0.8252	
Opisthokonta	*Nucletmycea*	*Fungi*	*Incertae Sedis*	*Paramicrosporidium*	0.07%	0.03%	0.00%	0.00%	0.2338	0.8252	
SAR	*Rhizaria*	*Cercozoa*	*Incertae Sedis*	*Gymnophrys*	0.02%	0.01%	0.00%	0.00%	0.4800	1	
Excavata	*Discoba*	*Jakobida*	*metagenome*	*metagenome*	0.01%	0.01%	0.00%	0.00%	0.4813	1	
Opisthokonta	*Nucletmycea*	*Fungi*	*Pichiaceae*	*Pichia*	0.05%	0.03%	0.00%	0.00%	0.4821	1	
Opisthokonta	*Nucletmycea*	*Fungi*	*Ophiostomataceae*	*NA*	0.02%	0.01%	0.00%	0.00%	0.4827	1	
Opisthokonta	*Nucletmycea*	*Fungi*	*Ustilaginaceae*	*NA*	0.01%	0.01%	0.00%	0.00%	0.4842	1	
Opisthokonta	*Nucletmycea*	*Fungi*	*Incertae Sedis*	*Wallemia*	0.08%	0.04%	0.00%	0.00%	0.4857	1	
Opisthokonta	*Nucletmycea*	*Fungi*	*Entomophthoraceae*	*NA*	0.03%	0.02%	0.00%	0.00%	0.4895	1	
Opisthokonta	*Nucletmycea*	*Fungi*	*Entomophthoraceae*	*Pandora*	0.01%	0.01%	0.00%	0.00%	0.4904	1	
Opisthokonta	*Holozoa*	*Choanoflagellida*	*Craspedida*	*uncultured*	0.02%	0.01%	0.00%	0.00%	1	1	
SAR	*Stramenopiles*	*Bicosoecida*	*uncultured*	*uncultured microeukaryote*	0.00%	0.00%	0.00%	0.00%	1	1	
SAR	*Stramenopiles*	*Incertae Sedis*	*Blastocystis*	*Blastocystis sp. subtype 3*	2.05%	1.51%	0.00%	0.00%	1	1	
Incertae Sedis	*Ancyromonadida*	*Nutomonas*	*Nutomonas longa*	*Nutomonas longa*	0.00%	0.00%	0.00%	0.00%	1	1	
Excavata	*Discoba*	*Discicristata*	*Tetramitia*	*Learamoeba*	0.00%	0.00%	0.00%	0.00%	1	1	
Excavata	*Discoba*	*Discicristata*	*Tetramitia*	*Allovahlkampfia*	0.00%	0.00%	0.00%	0.00%	1	1	
SAR	*Stramenopiles*	*Incertae Sedis*	*Blastocystis*	*Blastocystis sp. M5*	0.01%	0.01%	0.00%	0.00%	1	1	
SAR	*Rhizaria*	*Cercozoa*	*NA*	*NA*	0.01%	0.01%	0.00%	0.00%	1	1	

Phylum	Class	Order	Family	Genus	Mean$_{Ctrl}$	SE$_{Ctrl}$	Mean$_{PD}$	SE$_{PD}$	P	P$_{FDR}$	
SAR	*Alveolata*	*Apicomplexa*	*Eugregarinorida*	*NA*	0.01%	0.01%	0.00%	0.00%	1	1	
SAR	*Alveolata*	*Apicomplexa*	*Eugregarinorida*	*Gregarina*	0.01%	0.01%	0.00%	0.00%	1	1	
SAR	*Alveolata*	*Apicomplexa*	*Eugregarinorida*	*Leidyana*	0.02%	0.01%	0.00%	0.00%	1	1	
Opisthokonta	*Holozoa*	*Metazoa (Animalia)*	*Ellipura*	*Collembola*	0.01%	0.01%	0.00%	0.00%	1	1	
Opisthokonta	*Holozoa*	*Metazoa (Animalia)*	*Oligochaeta*	*Haplotaxida*	0.01%	0.01%	0.00%	0.00%	1	1	
SAR	*NA*	*NA*	*NA*	*NA*	0.02%	0.01%	0.00%	0.00%	1	1	
SAR	*Alveolata*	*Ciliophora*	*Platyophryida*	*Sorogena*	0.06%	0.05%	0.00%	0.00%	1	1	
SAR	*Alveolata*	*Ciliophora*	*Colpodida*	*Colpoda*	0.03%	0.03%	0.00%	0.00%	1	1	
SAR	*Alveolata*	*Ciliophora*	*Colpodea*	*Cyrtolophosidida*	0.00%	0.00%	0.00%	0.00%	1	1	
SAR	*Stramenopiles*	*Peronosporomycetes*	*NA*	*NA*	0.02%	0.02%	0.00%	0.00%	1	1	
Amoebozoa	*Discosea*	*Flabellinia*	*Vannellida*	*Vannella*	0.02%	0.01%	0.00%	0.00%	1	1	
SAR	*Alveolata*	*Dinoflagellata*	*NA*	*NA*	0.01%	0.01%	0.00%	0.00%	1	1	
Opisthokonta	*Nucletmycea*	*Fungi*	*Powellomycetaceae*	*NA*	0.01%	0.01%	0.00%	0.00%	1	1	
SAR	*Alveolata*	*Apicomplexa*	*Neogregarinorida*	*NA*	0.01%	0.01%	0.00%	0.00%	1	1	
Opisthokonta	*Nucletmycea*	*Fungi*	*LKM15*	*uncultured alveolate*	0.01%	0.01%	0.00%	0.00%	1	1	
Opisthokonta	*Nucletmycea*	*Fungi*	*Syncephalastraceae*	*Syncephalastrum*	0.01%	0.01%	0.00%	0.00%	1	1	
Opisthokonta	*Nucletmycea*	*Fungi*	*Tilletiaceae*	*NA*	0.01%	0.01%	0.00%	0.00%	1	1	
Opisthokonta	*Nucletmycea*	*Fungi*	*Pterulaceae*	*Pterula*	0.01%	0.00%	0.00%	0.00%	1	1	
Opisthokonta	*Nucletmycea*	*Fungi*	*Geastraceae*	*Sphaerobolus*	0.02%	0.01%	0.00%	0.00%	1	1	
Opisthokonta	*Nucletmycea*	*Fungi*	*Mrakiaceae*	*NA*	0.01%	0.01%	0.00%	0.00%	1	1	

Phylum	Class	Order	Family	Genus	Mean$_{Ctrl}$	SE$_{Ctrl}$	Mean$_{PD}$	SE$_{PD}$	P	P$_{FDR}$	
Opisthokonta	*Nucletmycea*	*Fungi*	*Incertae Sedis*	*NA*	0.01%	0.01%	0.00%	0.00%	1	1	
Opisthokonta	*Nucletmycea*	*Fungi*	*Dipodascaceae*	*Yarrowia*	0.01%	0.01%	0.00%	0.00%	1	1	
Opisthokonta	*Nucletmycea*	*Fungi*	*Phaffomycetaceae*	*Cyberlindnera-Candida clade*	0.02%	0.02%	0.00%	0.00%	1	1	
Opisthokonta	*Nucletmycea*	*Fungi*	*Saccharomycodaceae*	*Hanseniaspora*	0.04%	0.03%	0.00%	0.00%	1	1	
Opisthokonta	*Nucletmycea*	*Fungi*	*Trigonopsidaceae*	*Trigonopsis*	0.01%	0.01%	0.00%	0.00%	1	1	
Opisthokonta	*Nucletmycea*	*Fungi*	*Apiosporaceae*	*Arthrinium*	0.04%	0.03%	0.00%	0.00%	1	1	
Opisthokonta	*Nucletmycea*	*Fungi*	*Niessliaceae*	*Niesslia*	0.01%	0.00%	0.00%	0.00%	1	1	
Opisthokonta	*Nucletmycea*	*Fungi*	*Bionectriaceae*	*Geosmithia*	0.00%	0.00%	0.00%	0.00%	1	1	
Opisthokonta	*Nucletmycea*	*Fungi*	*Eremascaceae*	*Eremascus*	0.04%	0.03%	0.00%	0.00%	1	1	
Opisthokonta	*Nucletmycea*	*Fungi*	*Arthrodermataceae*	*NA*	0.01%	0.01%	0.00%	0.00%	1	1	
Opisthokonta	*Nucletmycea*	*Fungi*	*Taphrinaceae*	*Taphrina*	0.01%	0.00%	0.00%	0.00%	1	1	
Opisthokonta	*Nucletmycea*	*Fungi*	*Herpotrichiellaceae*	*NA*	0.02%	0.01%	0.00%	0.00%	1	1	
SAR	*Rhizaria*	*Cercozoa*	*Incertae Sedis*	*Kraken*	0.03%	0.02%	0.00%	0.00%	1	1	
SAR	*Rhizaria*	*Cercozoa*	*uncultured*	*uncultured eukaryote*	0.03%	0.02%	0.00%	0.00%	1	1	
SAR	*Rhizaria*	*Cercozoa*	*Cercomonadidae*	*uncultured Cercomonas*	0.01%	0.01%	0.00%	0.00%	1	1	
Archaeplastida	*Chloroplastida*	*Chlorophyta*	*Chlamydomonadales*	*NA*	0.02%	0.01%	0.00%	0.00%	1	1	
Archaeplastida	*Chloroplastida*	*Chlorophyta*	*Chlamydomonadales*	*Tetracystis*	0.00%	0.00%	0.00%	0.00%	1	1	
Archaeplastida	*Chloroplastida*	*Chlorophyta*	*Chlorophyceae*	*Chlamydomonadaceae sp. Pic8/18P-5w*	0.01%	0.01%	0.00%	0.00%	1	1	
Archaeplastida	*Chloroplastida*	*Chlorophyta*	*Chlorophyceae*	*Chlorococcales sp. VII3*	0.02%	0.01%	0.00%	0.00%	1	1	
Archaeplastida	*Chloroplastida*	*Chlorophyta*	*Trebouxiophyceae*	*NA*	0.02%	0.01%	0.00%	0.00%	1	1	

Phylum	Class	Order	Family	Genus	$Mean_{Ctrl}$	SE_{Ctrl}	$Mean_{PD}$	SE_{PD}	P	P_{FDR}	
Archaeplastida	*Chloroplastida*	*Charophyta*	*Malpighiales*	*Linum*	0.02%	0.02%	0.00%	0.00%	1	1	
SAR	*Stramenopiles*	*NA*	*NA*	*NA*	0.00%	0.00%	0.00%	0.00%	1	1	
